# Association between intensive care unit nursing grade and mortality in patients with cardiogenic shock and its cost-effectiveness

**DOI:** 10.1186/s13054-024-04880-9

**Published:** 2024-03-25

**Authors:** Ki Hong Choi, Danbee Kang, Jin Lee, Hyejeong Park, Taek Kyu Park, Joo Myung Lee, Young Bin Song, Joo-Yong Hahn, Seung-Hyuk Choi, Hyeon-Cheol Gwon, Juhee Cho, Jeong Hoon Yang

**Affiliations:** 1 Division of Cardiology, Department of Internal Medicine, Heart Vascular Stroke Institute, Samsung Medical Center, Sungkyunkwan University School of Medicine, 81 Irwon-ro, Gangnam-gu, Seoul, 06351 Republic of Korea; 2 Department of Clinical Research Design and Evaluation, SAIHST, Sungkyunkwan University, Seoul, Republic of Korea; 3 Center for Clinical Epidemiology, Samsung Medical Center, Sungkyunkwan University School of Medicine, 81 Irwon-ro, Gangnam-gu, Seoul, 06351 Republic of Korea; 4 Department of Critical Care Medicine, Samsung Medical Center, Sungkyunkwan University School of Medicine, Seoul, Republic of Korea

**Keywords:** Cardiogenic shock, Intensive care unit, Nurse staffing, Mortality, Cost-effectiveness

## Abstract

**Background:**

Despite the high workload of cardiac intensive care unit (ICU), there is a paucity of evidence on the association between nurse workforce and mortality in patients with cardiogenic shock (CS). This study aimed to evaluate the prognostic impact of the ICU nursing grade on mortality and cost-effectiveness in CS.

**Methods:**

A nationwide analysis was performed using the K-NHIS database. Patients diagnosed with CS and admitted to the ICU at tertiary hospitals were enrolled. ICU nursing grade was defined according to the bed-to-nurse ratio: grade1 (bed-to-nurse ratio < 0.5), grade2 (0.5 ≤ bed-to-nurse ratio < 0.63), and grade3 (0.63 ≤ bed-to-nurse ratio < 0.77) or above. The primary endpoint was in-hospital mortality. Cost-effective analysis was also performed.

**Results:**

Of the 72,950 patients with CS, 27,216 (37.3%) were in ICU nursing grade 1, 29,710 (40.7%) in grade 2, and 16,024 (22.0%) in grade ≥ 3. The adjusted-OR for in-hospital mortality was significantly higher in patients with grade 2 (grade 1 vs. grade 2, 30.6% vs. 37.5%, adjusted-OR 1.14, 95% CI1.09–1.19) and grade ≥ 3 (40.6%) with an adjusted-OR of 1.29 (95% CI 1.23–1.36) than those with grade 1. The incremental cost-effectiveness ratio of grade1 compared with grade 2 and ≥ 3 was $25,047/year and $42,888/year for hospitalization and $5151/year and $5269/year for 1-year follow-up, suggesting that grade 1 was cost-effective. In subgroup analysis, the beneficial effects of the high-intensity nursing grade on mortality were more prominent in patients who received CPR or multiple vasopressors usage.

**Conclusions:**

For patients with CS, ICU grade 1 with a high-intensity nursing staff was associated with reduced mortality and more cost-effectiveness during hospitalization compared to grade 2 and grade ≥ 3, and its beneficial effects were more pronounced in subjects at high risk of CS.

**Graphical abstract:**

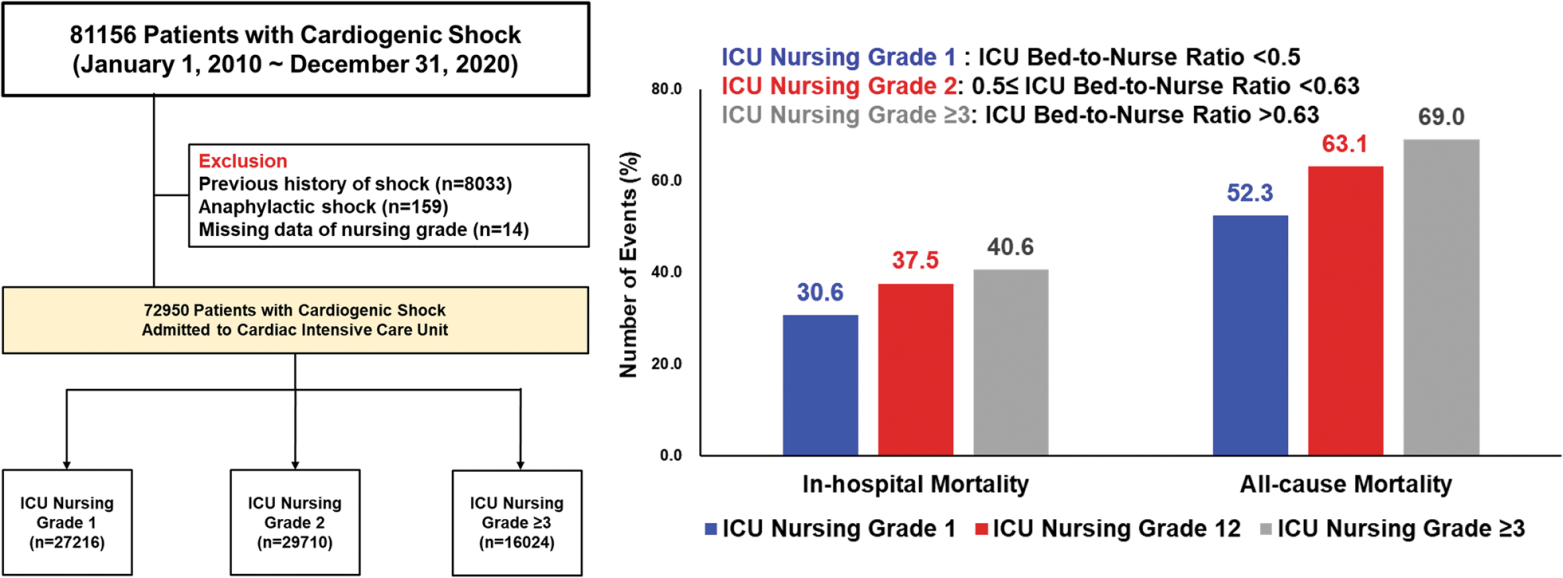

**Supplementary Information:**

The online version contains supplementary material available at 10.1186/s13054-024-04880-9.

## Introduction

Cardiogenic shock (CS) is defined as reduced cardiac output together with the impairment of multi-organ blood flow and oxygen delivery to meet metabolic demands [1, 2]. Although the widespread adoption of early revascularization strategies and enhancement of evidence-based medical treatments have led to a substantial decrease in mortality for patients with CS over the last 2 decades [3–7], refractory CS remains the leading cause of death worldwide. Recently, standardized CS management protocols—including the timely recognition of CS, hemodynamic monitoring, and tailored application of mechanical circulatory support (MCS)—have been emphasized to reduce mortality in patients with CS [8, 9]. However, variations in practice patterns for CS management across hospitals endure due to the significant human resources required to apply this careful approach.

Limited nursing staff is one of the challenges in caring for CS because nurses are designated individuals who are the first to detect the deterioration of a patient’s condition through close monitoring, which is the core of critical care [10]. Studies have found that the nurse staffing ratio is associated with hospitals’ quality of care and safety [11, 12]. Overworked nurses would have difficulties providing optimal patient care, leading to adverse outcomes and potentially affecting patients’ survival [13–15]. While extensive research has explored the general implications of nursing workload in critical care settings, there remains a paucity of data specifically focused on the unique challenges and outcomes associated with CS. Previous studies have often centered on broader ICU populations or specific surgical outcomes, such as those following esophageal surgery, which do not adequately capture the acute, life-threatening conditions inherent in CS [10, 16, 17]. This distinction underscores the necessity of our study, which concentrates on this underrepresented group within cardiac ICU settings. Considering the time-sensitive nature of CS, the negative impact of limited nurse staffing resources on the increased risk of death might be more pronounced in cardiac intensive care unit (ICU) settings [18]. In this regard, the American Heart Association proposes the categorization of cardiac ICU according to specific capabilities, including nurse staffing ratios, ranging from Level I (highest category) to III (lowest category), and care for patients at each level [19]. Nevertheless, many ICUs do not meet the recommended staffing standards, even in developed countries [20], which might affect clinical outcomes. Thus, we aimed to evaluate the association between ICU nursing grade and mortality in patients with CS using a national data. Given that nurse staffing accounts for a significant portion of hospital costs, we also conducted a cost-effectiveness analysis to assess the appropriate number of nurses in the cardiac ICU for managing patients with CS.

## Methods

### Study setting and data sources

This is a retrospective cohort study. We obtained data from the Korean National Health Insurance Service (K-NHIS) database. The K-NHIS covers approximately 97% of Koreans, whereas the remaining 3% of Koreans who cannot afford national insurance are covered by the Medical Aid Program [21]. Therefore, the K-NHIS database represents the entire population of South Korea and contains national records of all covered inpatient and outpatient visits, procedures, and prescriptions. The NHIS data includes modules on insurance eligibility and medical treatment. The insurance eligibility module contains information on age, sex, residential area, and income level. The medical treatment module contains information on treatment, including diseases and prescriptions [22].

For this study, we included patients aged ≥ 18 years who were diagnosed with cardiogenic shock and admitted to the ICU at tertiary hospitals between January 1, 2010, and December 31, 2020 (*N* = 81,156). Patients with cardiogenic shock were defined as having been admitted to the ICU with the diagnostic code for cardiogenic shock (International Classification of Disease, 10th revision codes: R57.0) and the presence of at least one of the following conditions: (1) presence of extracorporeal membrane oxygenation (ECMO) procedure code (K-NHIS procedure codes: O1901-O1904) or intra-aortic balloon pump (IABP, K-NHIS procedure codes: O1921, O1922); (2) prescription of vasoactive drugs including dopamine, norepinephrine, epinephrine, and vasopressin for at least 1 day and presence of mechanical ventilation (K-NHIS procedure codes: M5857, M5858, and M5860) or continuous renal replacement therapy (CRRT, K-NHIS procedure codes: O7031-O7034, O7051-O7054); or (3) prescription of a vasoactive drug for at least 2 days. Among the participants, we excluded those with anaphylaxis, as epinephrine can be used to treat it (codes: T78.0–T78.4, *N* = 159), and those with a history of shock (*N* = 8033). We also excluded participants with “missing” on the nursing grade (*N* = 14). The final sample size included 72,950 patients. The study was approved by the Institutional Review Board of Samsung Medical Center (2021-08-147) and informed consent was exempted because we only accessed de-identified data.

### Measurement

ICU nursing grade was defined using codes for ICU nursing-grade admission fees (K-NHIS procedure codes: AJ11–AJ19, AJ21–AJ29, and AJ31–AJ39), which represent the ratio of the number of beds to nurses. ICU nursing grades were categorized into nine (grades 1–9): grade 1 (the best) indicated that the ratio of the number of beds per nurse was less than 0.5, and grade 9 (the worst) indicated that the ratio was ≥ 2.5. For this study, we made three groups according to the ICU nursing grade: grade 1 (bed-to-nurse ratio < 0.5), grade 2 (0.5 ≤ bed-to-nurse ratio < 0.63), and grade 3 (0.63 ≤ bed-to-nurse ratio < 0.77) or above.

In-hospital mortality was defined as the receipt of an insurance death code at index admission. All-cause mortality was defined as the receipt of an insurance death code during the follow-up period (until December 31, 2020).

Information on the underlying disease, interventions, demographics, and hospital characteristics was based on claim codes. We used the Korean Classification of Disease, sixth edition, which is a modified version of the International Classification of Disease, 10th revision adapted for use in the Korean health system [23]. Cardiopulmonary resuscitation (CPR) at admission was defined as the presence of a CPR procedure code (K-NHIS procedure codes: M5871, M5873, M5874, M5875, M5876, and M5877) in the emergency room prior to admission for the patients admitted from the emergency room. Interventions for critical care included the use of mechanical ventilation for more than 3 h (K-NHIS procedure codes: M5857, M5858, and M5860), ECMO (O1901–O1904), hemodialysis including CRRT (O7051-O7054), hemodialysis (O7020), and peritoneal dialysis (O7062). We identified the use of vasopressor drugs such as dobutamine (Anatomical Therapeutic Chemical [ATC] code: C01CA07), dopamine (ATC code: C01CA04), epinephrine (ATC code: C01CA24), and norepinephrine (ATC code: C01CA03) for more than 2 days using Korean drugs and anatomical therapeutic chemical codes. We calculated hospital volumes using the number of patients per year. We also collected the total medical costs during the index hospitalization.

### Statistical analysis

Chi-square and Student’s t-tests were used to compare categorical and continuous variables, respectively. The primary outcome of this study was in-hospital mortality. We calculated odds ratios (ORs) with 95% confidence intervals (CIs) for in-hospital mortality using logistic regression. We adjusted for age, sex, cause of admission, comorbidities, use of multiple vasopressors, mechanical ventilation, IABP, ECMO, CRRT, and hospital volume to account for potential confounding factors. To account for potential non-linear associations between continuous covariates and outcomes, age and hospital volume included in the model using restricted cubic splines with 4 knots (solid curve). According to the guideline for improving causal inference, covariables were selected a priori based on their possible associations with ICU nursing grade and outcomes [24]. In addition, we explored the association between the ratio of the number of beds to nurses and in-hospital mortality in pre-specified, clinically relevant subgroups defined by age, sex, CPR at admission, multiple vasopressor use, mechanical ventilation, and ECMO. For all-cause mortality, patients were followed from the date of admission until the date of death or December 31, 2020 (end of the study), whichever occurred first. Cumulative all-cause mortality during the entire study period was estimated using the Kaplan–Meier method, and log-rank tests were used to evaluate the differences between the groups. We calculated the hazard ratios (HRs) with a 95% CI for all-cause mortality using a Cox regression model. We examined the proportional hazard assumption using plots of the log–log survival function and Schoenfeld residuals.

A cost-effectiveness analysis was conducted followed the Consolidated Health Economic Evaluation Reporting Standards guidelines [25]. A time horizon of in-hospital (from admission to discharge) and 1 year was applied. We estimated the length of survival from the time of admission to all-cause mortality and calculated the daily medical costs based on index hospitalization. In the analysis, the cost was presented in US dollars (1200 Korean Won [₩] = 1 dollar [$]). The cost-effectiveness of nursing grade 1 was expressed as the incremental cost-effectiveness ratio (ICER), defined as the difference in the in-hospitalization costs divided by the difference in length of survivor within hospitalization and 1 year after discharge. To determine the optimal strategies, we utilized a willingness to pay threshold of $45,000, which is considered severe for ill conditions. We considered interventions with ICER values lower than the 1 × willingness to pay threshold to be cost-effective. A strategy that was both less costly and more effective than another was defined as dominant [26]. We also performed a probabilistic sensitivity analysis. The incremental cost-effectiveness plane for differences in costs and quality-adjusted life years and ICER was obtained using the bootstrap technique with the percentile method with 25,000 replications. Planned subgroup analyses of the cost-effectiveness of grade 1 of the ratio of the number of beds to nurses were performed according to CPR at admission, use of multiple vasopressors, mechanical ventilation, and ECMO.

All *p* values were two-sided, and a *p* value < 0.05 was considered significant. Analyses were performed using SAS® (SAS Institute Inc., USA) and R 4.1.2 (R Foundation for Statistical Computing, Vienna, Austria).

## Results

### Patient characteristics

The participants’ mean (standard deviation) age was 69.4 (14.4) years, and 60.1% of them were male (Table 1). Of these, 33.2% visited hospitals due to acute myocardial infarction-related CS, and the remaining 66.8% visited due to non-acute myocardial infarction-related CS. Out of these patients, 63.4% required mechanical ventilation, and 21.3% and 8.0% of patients required CRRT and ECMO, respectively. In terms of nursing grading, the participants were divided as 37.3% (*N* = 27,216) in ICU grade 1, 40.7% (*N* = 29,710) in ICU grade 2, and 22.0% (*N* = 16,024) in ICU grade ≥ 3. In comparison to patients in ICU grade 2 or grade ≥ 3, patients in ICU grade 1 had higher general risk factors, such as Charlson’s comorbidity index, history of congestive heart failure, diabetes mellitus, chronic kidney disease, and history of cancer. In addition, patients in ICU grade 1 were more likely to require ECMO (9.6% vs. 7.3% vs. 6.8%; *p* < 0.01) and CRRT (22.6% vs. 22.0% vs. 17.7%; *p* < 0.01; Table 1).

**Table 1:** Characteristics of patients with cardiogenic shock by ICU nursing grade

	Overall	ICU nursing grade 1	ICU nursing grade 2	ICU nursing grade ≥ 3	*p* value
	(N = 72,950)	(N = 27,216)	(N = 29,710)	(N = 16,024)	
Age, mean (SD)	69.4 (14.4)	69.3 (14.5)	69.5 (14.5)	69.5 (14.0)	0.19
Sex, male	43,844 (60.1)	16,885 (62.0)	17,725 (59.7)	9234 (57.6)	< 0.001
Charlson’s index, mean (SD)	3.4 (2.8)	3.5 (2.8)	3.4 (2.8)	3.1 (2.7)	< 0.001
Medical aid, yes	5092 (7.0)	1393 (5.1)	2238 (7.5)	1461 (9.1)	< 0.001
History of myocardial infarction	10,188 (14.0)	3741 (13.8)	4054 (13.7)	2393 (14.9)	< 0.001
History of congestive heart failure	23,203 (31.8)	8997 (33.1)	9505 (32.0)	4701 (29.3)	< 0.001
Diabetes mellitus	31,167 (42.7)	11,878 (43.6)	12,749 (42.9)	6540 (40.8)	0.21
Hypertension	42,213 (57.9)	15,716 (57.8)	17,439 (58.7)	9058 (56.5)	0.002
Chronic kidney disease	10,358 (14.2)	4382 (16.1)	4197 (14.1)	1779 (11.1)	0.002
History of cancer	7676 (10.5)	3560 (13.1)	2797 (9.4)	1319 (8.2)	0.09
Cause of admission					< 0.001
AMI-CS	24,214 (33.2)	8834 (32.5)	9624 (32.4)	5756 (35.9)	
Non-AMI-CS	48,736 (66.8)	18,382 (67.5)	20,086 (67.6)	10,268 (64.1)	
Admission from emergency room	61,812 (84.7)	22,063 (81.1)	25,770 (86.7)	13,979 (87.2)	< 0.001
CPR at admission	13,747 (18.8)	2942 (10.8)	6235 (21.0)	4,70 (28.5)	< 0.001
Use of vasopressor drugs					
Dopamine	44,688 (61.3)	13,345 (49.0)	18,269 (61.5)	13,074 (81.6)	< 0.001
Norepinephrine	53,796 (73.7)	21,007 (77.2)	22,766 (76.6)	10,023 (62.6)	< 0.001
Epinephrine	34,058 (46.7)	11,762 (43.2)	14,443 (48.6)	7853 (49.0)	< 0.001
Vasopressin	11,339 (15.5)	5426 (19.9)	4549 (15.3)	1364 (8.5)	< 0.001
Multiple vasopressors	43,400 (59.5)	14,881 (54.7)	18,425 (62.0)	10,094 (63.0)	< 0.001
Concomitant use of Inotropes	23,888 (32.7)	8478 (31.2)	9747 (32.8)	5663 (35.3)	< 0.001
Mechanical ventilation	46,254 (63.4)	15,934 (58.6)	19,587 (65.9)	10,733 (67.0)	< 0.001
ECMO	5867 (8.0)	2620 (9.6)	2158 (7.3)	1089 (6.8)	< 0.001
IABP	3863 (5.3)	1359 (5.0)	1384 (4.7)	1120 (7.0)	< 0.001
CRRT	15,532 (21.3)	6158 (22.6)	6540 (22.0)	2834 (17.7)	< 0.001
Length of stay (days)	20.7 (44.8)	22.4 (55.8)	20.3 (37.6)	18.8 (34.8)	< 0.001
Hospital volume	187.4 (128.2–224.1)	220.3 (155.1–238.7)	157.7 (106.9–199.3)	155.1 (106.9–190.7)	< 0.001

Values were presented as n (%), mean (SD), or median (interquartile range)

*AMI-CS* acute myocardial infarction-related cardiogenic shock, *CRRT* continuous renal replacement therapy, *CPR* cardio-pulmonary resuscitation, *ECMO* extracorporeal membrane oxygenation, *IABP* intra-aortic balloon pump, *ICU* intensive care unit, *SD* standard deviation

### In-hospital and follow-up outcomes

**Fig. 1:**
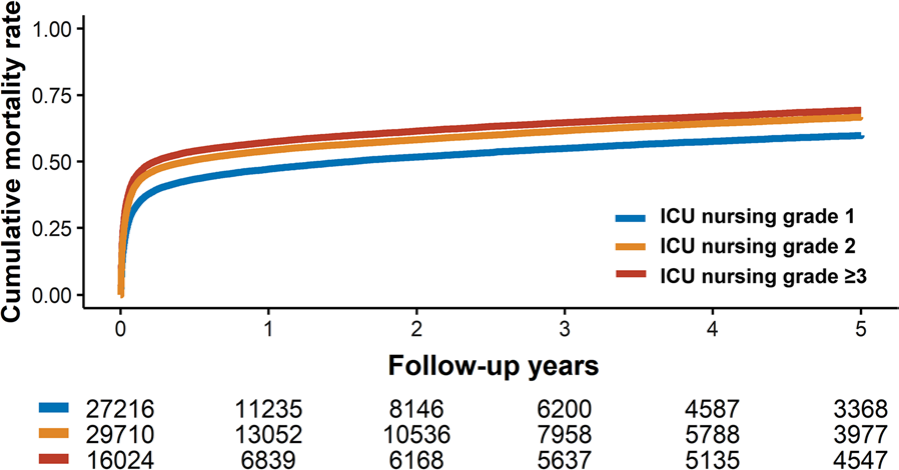
Comparison of all-cause mortality according to ICU nursing grade in patients with CS. Kaplan–Meier curves are shown for comparing 5-year follow-up, all-cause mortality among patients treated by ICU nursing grade 1 (blue line), grade 2 (orange line), or grade ≥ 3 (red line) hospitals. *CS* cardiogenic shock, *ICU* intensive care unit

The in-hospital mortality rates for patients in ICU grade 1, ICU grade 2, and ICU grade ≥ 3 groups were 30.6%, 37.5%, and 40.6%, respectively. The fully-adjusted ORs for in-hospital mortality in patients with grade 2 and grade ≥ 3, compared to those with grade 1, were 1.14 (95% CI 1.09–1.19) and 1.29 (95% CI1.23–1.36), respectively (Table 2). During the median 0.5 years of follow-up (interquartile range 0.03–3.41, maximum 11 years), 44,046 participants died. The mortality rate per 100 person-years was 52.3, 63.1 and 69.0 in ICU grades 1, 2, and ≥ 3 (Fig. 1, log-rank *p* < 0.01). Crude HR (95% CI) for all-cause mortality was 1.23 and 1.34 when comparing grades 2 and ≥ 3 to grade 1 (Table 2). This association remained significant after adjusting for confounding factors (grade 1 vs. 2, HR 1.10, 95% CI 1.07–1.13; grade 1 vs. grade ≥ 3, HR 1.21, 95% CI 1.18–1.24).

**Table 2:** In-hospital and all-cause mortality among patients with cardiogenic shock by ICU nursing grade

	ICU nursing grade 1 (N = 27,216)	ICU nursing grade 2 (N = 29,710)	ICU nursing grade ≥ 3 (N = 16,024)
In-hospital mortality			
No. of events (%)	8,321 (30.6)	11,154 (37.5)	6,506 (40.6)
Crude OR (95% CI)	Reference	**1.37 (1.32–1.41)**	**1.55 (1.49–1.62)**
Adjusted OR* (95% CI)	Reference	**1.14 (1.09–1.19)**	**1.29 (1.23–1.36)**
All-cause mortality			
No. of events (% per patient-year)	14,240 (52.3)	18,747 (63.1)	11,059 (69.0)
Crude HR (95% CI)	Reference	**1.23 (1.20–1.26)**	**1.34 (1.31–1.37)**
Adjusted HR (95% CI)	Reference	**1.10 (1.07–1.13)**	**1.21 (1.18–1.24)**

Statistically significant results (P < 0.05) were highlighted in bold

*CI* confidence interval, *OR* odds ratio, *CRRT* continuous renal replacement therapy, *CPR* cardiopulmonary resuscitation, *ECMO* extracorporeal membrane oxygenation, *HR* hazard ratio, *IABP* intra-aortic balloon pump

*Models adjusted for age, sex, cause of admission, comorbidities, multiple vasopressors, CPR, mechanical ventilation, IABP, ECMO, CRRT, and hospital volume

In the subgroup analysis, increased in-hospital mortality in patients with ICU grade 2 compared with those with ICU grade 1 was more prominent in patients who received CPR on admission (*P*-for-interaction < 0.01) than in those who did not receive it. In ICU grade ≥ 3 versus ICU grade 1, the effect of increased mortality was stronger in patients who received CPR on admission (*P*-for-interaction < 0.01) and multiple vasopressors (*P*-for-interaction < 0.01) than in those who did not receive CPR or received a single vasopressor (Additional file 1: Fig. S1).

### Cost-effectiveness

**Fig. 2:**
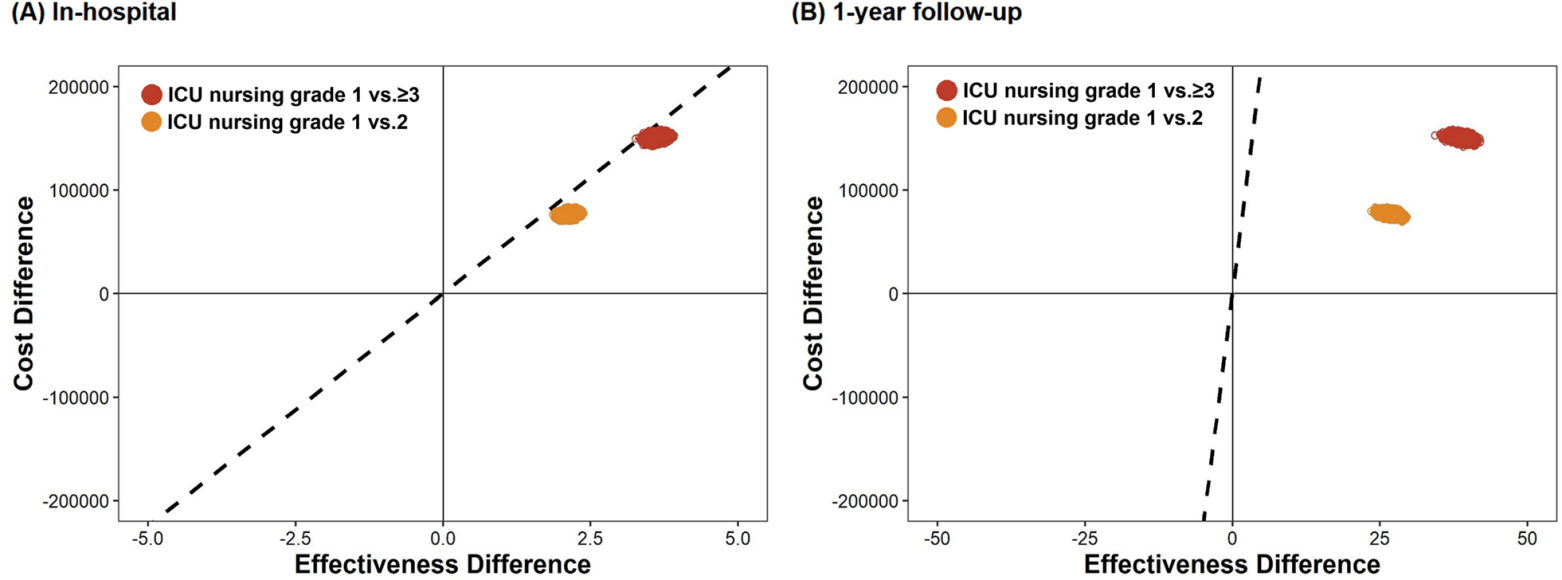
Incremental cost-effectiveness plane for ICU nursing grade 1 compared with grade 2 and grade ≥ 3 for costs until discharge and 1 year after admission. Replications of the incremental cost-effectiveness of ICU grade 1 compared with ICU grade 2 (orange dot) and ICU grade ≥ 3 (red dot) are shown. The incremental cost-effectiveness plane presented the effectiveness of admission to the grade 1 hospital for cardiogenic shock treatment, as compared to grades 2 and 3, on the difference in length of survival (effectiveness) and accompanying healthcare-related costs (dollars) during hospitalization and 1 year of follow-up. Each of the 25,000 points represents the result of one bootstrap replication. The difference in cumulative costs is displayed on the vertical axis, and the difference in the length of survival is displayed on the horizontal axis. The average ICER is indicated by a red dot. Willingness-to-pay thresholds of $45,000 per 1-year life gain added (dashed line) are indicated in the plane. *ICU* intensive care unit

The estimated total cumulative cost per patient for grade 1 was $1365—approximately $199 higher than in grade 2 and $423 higher than in grade ≥ 3. Comparing grade 1 with grade 2 and 3, we found that grade 1 patients lived for 2.9 and 3.6 days longer, respectively, and they lived an additional 14.1 and 29.3 days longer at the 1-year follow-up, respectively (Table 3 and Additional file 1: Table S1). The ICER comparing grade 2 and grade ≥ 3 was calculated as 25,047$/year and 42,888$/year for hospitalization and 5151$/year and 5269$/year for a 1-year follow-up period, respectively (Table 3). Grade 1 had higher cost-effectiveness across all analyzed subgroups, especially in patients who received CPR at admission, mechanical ventilation, and ECMO, than grades 2 and ≥ 3 (Table 3). Probabilistic sensitivity analysis showed that the probability of cost-effectiveness in grade 1 compared to grade 2 and in grade 1 compared to grade ≥ 3 was 100% and 99.9% in-hospital, and 100% and 100% at the 1-year follow-up, respectively (Fig. 2).

**Table 3:** Incremental Cost-Effectiveness Ratio (ICER) of ICU nursing grade 1

	ICU nusring grade 2 versus grade 1			ICU nursing grade ≥ 3 versus grade 1		
	Cost ($) Incremental	Length of survival (day) Incremental	ICER ($/year)*	Cost ($) Incremental	Length of survival (day) Incremental	ICER ($/year)*
In-hospital mortality						
Overall	199	2.9	25,047	423	3.6	42,888
CPR at admission						
No	248	1.8	50,289	511	1.2	155,429
Yes	93	1.4	24,246	304	2.5	44,384
Mechanical ventilation						
No	275	− 1.3	Dominated	490	− 1.6	Dominated
Yes	143	5.6	9321	372	6.8	19,968
ECMO						
No	215	1.8	43,597	425	2.4	64,635
Yes	55	13.3	1509	429	18.2	8604
CRRT						
No	233	0.4	212,613	438	1	159,870
Yes	73	11.1	2400	352	13.6	9447
1-year mortality						
Overall	199	14.1	5151	423	29.3	5269
CPR at admission						
No	248	7.5	12,069	511	16.7	11,169
Yes	93	4.1	8279	304	17.1	6489
Mechanical ventilation						
No	275	14.6	6875	490	24.3	7360
Yes	143	12.9	4046	372	30.7	4423
ECMO						
No	215	14.0	5605	425	28.6	5424
Yes	55	10.8	1859	429	32.6	4803

Model adjusted for age, sex, cause of admission, comorbidities, CPR on admission, multiple vasopressors, mechanical ventilation, IABP, ECMO, CRRT, and hospital volume

*CPR* cardiopulmonary resuscitation, *CRRT* continuous renal replacement therapy, *ECMO* extracorporeal membrane oxygenation, *ICER* incremental cost-effectiveness ratio

*1 US Dollar ($) = 1200 Won (₩); ICER($/year) = ICER($/Day) × 365

## Discussion

This large, nationwide cohort study suggests that a lower bed-to-nurse ratio is associated with decreased in-hospital mortality in patients with CS (Graphical Abstract). Furthermore, ICU nursing grade 1 was more cost-effective than grades 2 and ≥ 3 during hospitalization and 1-year follow-up. The association between the bed-to-nurse ratio and mortality benefit was more pronounced in patients who underwent CPR at admission and required multiple vasopressors, indicating Society for Cardiovascular Angiography and Interventions (SCAI) shock classification stage D or E. Finally, admission to an ICU grade 1 hospital seems to be more cost-effective when dealing with situations that require advanced resources and careful management, such as mechanical ventilation, ECMO, and CPR.

In the ICU setting, an increased nurse workload was associated with higher mortality rates [10, 27, 28]. In addition, the shortage of nursing staff can lead to problems of insufficient supervision, and it may inhibit the early recognition of patients’ changes.[29]. In this regard, the American Nurses Association recommends that critical care units have a patient-to-nurse ratio of 1:2 or less [30]. However, data comparing the clinical outcomes based on the ICU nursing grade for patients with CS, which is a life-threatening, acute condition, are limited. Theoretically, nursing staff resources are more critical in the setting of patients in time-sensitive situations such as shock and arrest than in the setting of elective care for monitoring high-risk patients [12]. Furthermore, CS is a complex condition that requires not only treatment of the heart but also prevention and treatment of the development of multiple organ dysfunction, along with the use of advanced mechanical circulatory devices [31]. Considering the potential impact of prolonged shock leading to systemic inflammatory response syndrome and multiple organ dysfunction syndrome on mortality rates [32, 33], enough nursing care to detect the early identification of potential complications is required to improve outcomes through continuous, careful monitoring in patients with CS. In particular, with the transformation from a conventional coronary care unit to a cardiac ICU, the focus on nursing workload and a multidisciplinary shock team approach in the setting of CS has been increasing [19, 34]. In fact, nurse-to-patient ratio is an important factor when evaluating cardiac ICU quality, and a level 1 cardiac ICU is defined as a nurse-to-patient ratio of 1:1 or 1:2 [19]. In line with this background, we found that hospitals with ICU nursing grade 2 (0.5 ≤ bed-to-nurse ratio < 0.63) and grade 3 (0.63 ≤ bed-to-nurse ratio < 0.77) or above showed significantly higher risks of mortality compared to hospitals with ICU nursing grade 1 (bed-to nurse ratio < 0.5), when treating patients with CS.

In our subgroup analysis, the beneficial effects of lower bed-to-nurse ratios on mortality were more prominent in patients classified as having an advanced form of CS requiring CPR at admission or multiple types of vasopressors to maintain tissue perfusion. Recently, the SCAI proposed a new 5-stage CS classification to provide a simple schema that would allow clear communication regarding patient status and allow clinical trials to appropriately differentiate patient subsets [35]. Several observational studies have clearly demonstrated that the SCAI shock stage is associated with robust mortality risk stratification in patients with CS [36–38]. Especially in the advanced stages of CS, such as SCAI D (multiple vasopressor use) or E (undergoing CPR at admission), patients might be highly susceptible to minor medical errors. In addition, inadequate physician collaboration, poor nurse–patient communication, increased medical errors, and nosocomial infections may be possible causes of the association between high nurse workload and increased mortality [27, 39]. Taken together, close monitoring, timely interventions, and efforts to reduce medical errors should be paid to rescue patients, particularly in the advanced form of CS, in accordance with the current findings.

The current study also showed that ICU grade 1 was more cost-effective than ICU grades 2 and 3 during hospitalization and at the 1-year follow-up. In the simplest scenario, increasing registered nurse staffing by 1 h per patient per day in the United Kingdom would cost $77,957 per life saved (2021 US dollar equivalent). Given a per capita gross domestic product of $47,334, this would be cost-effective if each life saved gained 1.6 quality-adjusted life years. Although the total cumulative cost per patient in grade 1 was higher than those in grades 2 and 3, it was still within the acceptable cost range, considering the significant improvement in life gain. The additional costs incurred in Grade 1 may have been offset by the benefits of extended survival and improved patient outcomes. In particular, patients who underwent invasive treatment with additional resources, such as CPR, mechanical ventilation, and ECMO, were more cost-effective in ICU grade 1 compared to the ICU grade 2 and grade ≥ 3 than those who did not. Considering the high cost of invasive procedures, it is essential to recognize that without adequate nurse staffing, expensive medical equipment may not yield the desired outcomes. Therefore, ensuring sufficient nursing resources is crucial to optimize the effectiveness of costly medical interventions in the care of patients with CS. Our findings have implications for clinical management and policymaking. Although we are aware that a high workload is associated with poor clinical outcomes [40], we could not assign a nurse to a patient according to the individual patient’s workload score before measuring individual patients. However, we could assume that when duties are assigned, patients require a high nurse staffing ratio and have priority in nurse allocation, which could be a cost-effective strategy for the management of patients with advanced CS.

### Limitations

This study has some limitations. First, the identification of CS is based on ICD-10 codes reported by hospitals. Given that the recent Extracorporeal Life Support Organization registry reported that only 50% of CS patients receiving ECMO support had an ICD code of R57.0 [41], there was a possibility that many patients with CS were missed in the current study. However, as recommended by the Shock Academic Research Consortium [42], we applied a strict additional definition of signs of organ failure, including mechanical ventilation, CRRT, IABP, or ECMO, and use of vasopressors for at least 2 days, to compensate for code-based sampling. Secondly, due to the nature of the dataset, we were not able to include certain key variables that could significantly influence the interpretation of our results. In particular, our dataset lacked direct measures of disease severity, such as multiple organ dysfunction syndrome, Acute Physiology and Chronic Health Evaluation, or Sequential Organ Failure Assessment scores, which are crucial for a comprehensive assessment of a patient’s clinical status. However, we attempted to adjust for severity of illness using the Shock Academic Research Consortium’s use of multiple vasopressors, CPR on admission, use of mechanical ventilation, IABP, ECMO, and CRRT as signs of organ failure. In addition, our dataset did not include information on the goals of care for individual patients, which can strongly influence treatment decisions and outcomes. Goals of care, which include critical decisions such as whether patients would accept CPR or intubation, play a key role in determining the intensity and type of care provided. Future studies incorporating these critical parameters would be necessary to better understand and improve patient care in cardiogenic shock scenarios. Thirdly, the measurement of nursing staffing levels was based on the number of hospital beds per nurse rather than patients per nurse. This approach was necessitated by the structure of our claims data, where nursing grades were determined based on the number of hospital beds at the time of data collection. Finally, our dataset lacked important information that could further influence patient outcomes, such as the scheduling of nursing shifts (morning, evening, or night) and the specific qualifications of nursing staff (e.g., nurse practitioner, registered nurse, licensed practical nurse, or certified nurse assistant).

## Conclusions

ICU grade 1 with a high-intensity nurse staffing was associated with reduced mortality and was more cost-effective during hospitalization and 1-year follow-up, compared to hospitals with ICU grade 2 and ≥ 3 in patients with CS. The beneficial effects of lower bed-to-nurse ratios were more pronounced in patients with an advanced form of CS who received CPR and multiple vasopressors.

### Supplementary Information

**Additional file 1.** Supplementary table (Table S1) and figure (Fig. S1).

## Data Availability

We used the claim data provided by the Korean National Health Insurance Service (NHIS) database. Data can only be accessed by visiting the NHIS datacenter, after approval from data access committee of NHIS. Those who want to access data set of this study should contact corresponding authors (jcho@skku.edu), who will help with the process of contacting the NHIS.

## Abbreviations

CPR: Cardiopulmonary resuscitation
CRRT: Continuous renal replacement therapy
CS: Cardiogenic shock
ECMO: Extracorporeal membrane oxygenation
ICER: Incremental cost-effectiveness ratio
ICU: Intensive care unit
K-NHIS: Korean National Health Insurance Service
MCS: Mechanical circulatory support
SCAI: Society for Cardiovascular Angiography and Interventions
