# Supplementary material for: Association between intensive care unit nursing grade and mortality in patients with cardiogenic shock and its cost-effectiveness
Source: Crit Care. 2024 Mar 25;28:99. doi: 10.1186/s13054-024-04880-9 (PMC10962168; doi:10.1186/s13054-024-04880-9)
Supplement: Supplementary file 1 — Additional file 1. Supplementary table (Table S1) and figure (Fig. S1). [file 13054_2024_4880_MOESM1_ESM.docx]

**SUPPLEMENTAL MATERIAL**

**Association between intensive care unit nursing grade and mortality in patients with cardiogenic shock and its cost-effectiveness**

Ki Hong Choi, MD, PhD; Danbee Kang; Juhee Cho; Jeong Hoon Yang, MD, PhD; et al.

**Table of Contents**

- **Supplementary Table 1**
- **Supplementary Figure 1 and Figure Legend**

**Supplementary Table 1. Adjusted cost and length of survival by ICU nursing grade**

|  | **Cost ($)**^*^ | | | **Length of survival (Days)** | | |
| --- | --- | --- | --- | --- | --- | --- |
|  | **ICU nursing grade 1** | **ICU nursing grade 2** | **ICU nursing grade ≥3** | **ICU nursing grade 1** | **ICU nursing grade 2** | **ICU nursing grade ≥3** |
| **In-hospital mortality** |  |  |  |  |  |  |
| **Overall** | 1,365 | 1,166 | 942 | 22.7 | 19.8 | 19.1 |
| **CPR** |  |  |  |  |  |  |
| No | 1,302 | 1,054 | 791 | 23.3 | 21.5 | 22.1 |
| Yes | 1,704 | 1,611 | 1,400 | 15.2 | 13.8 | 12.7 |
| **Mechanical ventilation** |  |  |  |  |  |  |
| No | 1,306 | 1,031 | 816 | 11.7 | 13.0 | 13.3 |
| Yes | 1,388 | 1,245 | 1,016 | 29.4 | 23.8 | 22.6 |
| **ECMO** |  |  |  |  |  |  |
| No | 1,229 | 1,014 | 804 | 21.3 | 19.5 | 18.9 |
| Yes | 2,950 | 2,895 | 2,521 | 37.1 | 23.8 | 18.9 |
| **1-year mortality** |  |  |  |  |  |  |
| **Overall** | 1,365 | 1,166 | 942 | 202.8 | 188.7 | 173.5 |
| **CPR** |  |  |  |  |  |  |
| No | 1,302 | 1,054 | 791 | 221.9 | 214.4 | 205.2 |
| Yes | 1,704 | 1,611 | 1,400 | 90.2 | 86.1 | 73.1 |
| **Mechanical ventilation** |  |  |  |  |  |  |
| No | 1,306 | 1,031 | 816 | 280.7 | 266.1 | 256.4 |
| Yes | 1,388 | 1,245 | 1,016 | 157.2 | 144.3 | 126.5 |
| **ECMO** |  |  |  |  |  |  |
| No | 1,229 | 1,014 | 804 | 209.1 | 195.1 | 180.5 |
| Yes | 2,950 | 2,895 | 2,521 | 128.0 | 117.2 | 95.4 |

Models adjusted for age, sex, cause of admission, comorbidities, CPR on admission, multiple vasopressors, mechanical ventilation, IABP, ECMO, CRRT, and hospital volume.

^*^1 US Dollar ($) = 1,200 Won (₩).

Abbreviations: CPR, cardiopulmonary resuscitation; CRRT, continuous renal replacement therapy; ECMO, extracorporeal membrane oxygenation.

**Supplementary Figure 1. Adjusted odds ratio for in-hospital mortality in ICU nursing grade 2 and grade ≥3 compared to ICU grade 1 by Subgroup.**


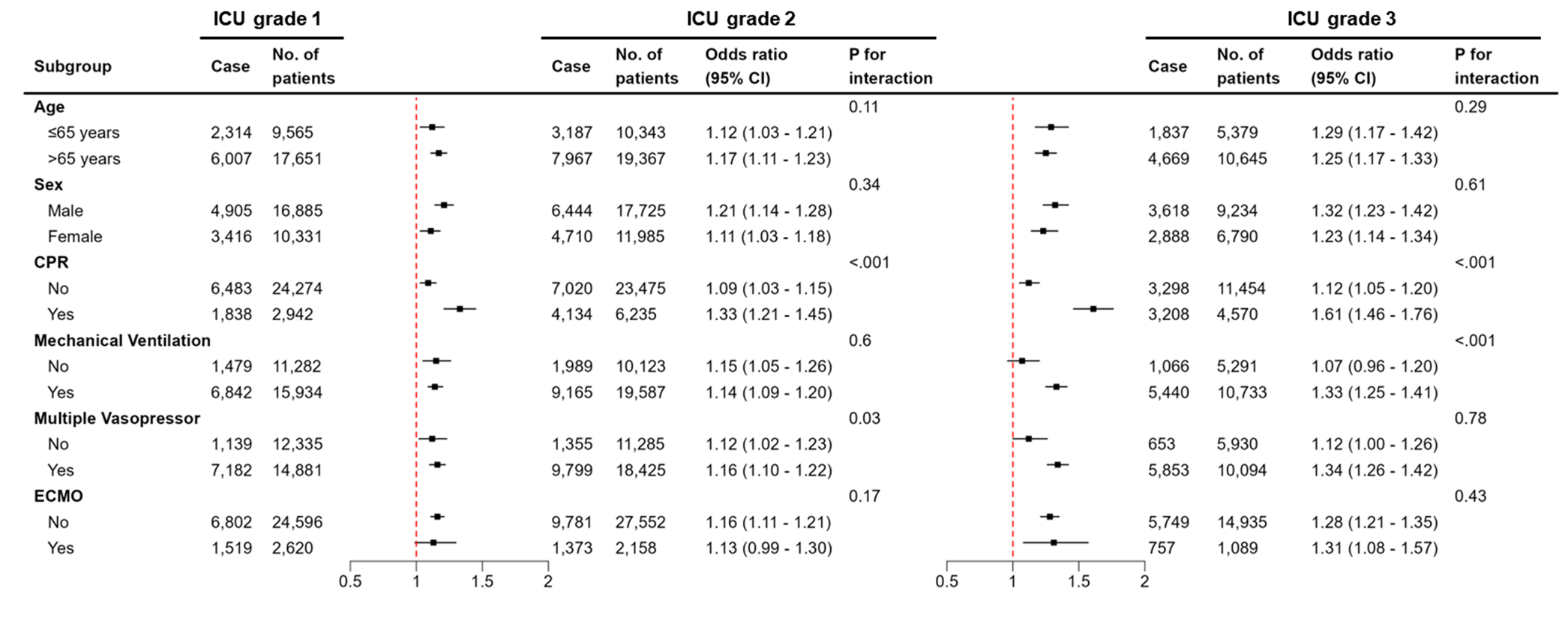


Models adjusted for age, sex, cause of admission, comorbidities, CPR on admission, multiple vasopressors, mechanical ventilation, IABP, ECMO, CRRT, and hospital volume.

Abbreviations: CI, confidence interval; CPR, cardiopulmonary resuscitation; CRRT, continuous renal replacement therapy; ECMO, extracorporeal membrane oxygenation; IABP, intra-aortic balloon pump; ICU, intensive care unit.
